# Combined effect of purulent vaginal discharge and anovulation on pregnancy status in a large multi-state population of Holstein cows

**DOI:** 10.3168/jdsc.2022-0271

**Published:** 2022-09-30

**Authors:** P. Pinedo, J.E.P. Santos, K.N. Galvão, G.M. Schuenemann, R.C. Chebel, R.C. Bicalho, R.O. Gilbert, S.L. Rodriguez-Zas, C.M. Seabury, G.J.M. Rosa, W.W. Thatcher

**Affiliations:** 1 Department of Animal Sciences, Colorado State University, Fort Collins 80521; 2 Department of Animal Sciences, University of Florida, Gainesville 32611; 3 College of Veterinary Medicine, University of Florida, Gainesville 32611; 4 Department of Veterinary Preventive Medicine, The Ohio State University, Columbus 43210; 5 College of Veterinary Medicine, Cornell University, Ithaca, NY 14850; 6 School of Veterinary Medicine, Ross University, St. Kitts, West Indies; 7 Department of Animal Sciences, University of Illinois, Urbana 61801; 8 College of Veterinary Medicine, Texas A&M University, College Station 77843; 9 Department of Animal and Dairy Sciences, University of Wisconsin, Madison 53706

## Abstract

The objective of this observational prospective cohort study was to evaluate the combined effect of purulent vaginal discharge (PVD) and anovulation (ANOV) on the reproductive performance of a large multi-state population of Holstein cows. Data were prospectively collected from 11,729 cows in 16 herds located in 4 regions in the United States [Northeast (4 herds), Midwest (6), Southeast (1), and Southwest (5)]. Cows were enrolled at calving and monitored weekly for disease occurrence, reproductive events, and survival. Prevalence of PVD was evaluated at 28 ± 3 d in milk and defined by the presence of mucopurulent to fetid vaginal discharge. Resumption of ovarian cyclicity was determined via transrectal ultrasonography at 40 ± 3 and 54 ± 3 d postpartum. Pregnancy diagnosis was performed by ultrasonography on d 32 ± 3 after artificial insemination (AI) and reconfirmed at d 60 ± 3 of gestation. Pregnancy loss (PL) was defined as a cow diagnosed pregnant at 32 ± 3 but nonpregnant at 60 ± 3 d after AI. The association of PVD and ANOV with pregnancy traits was analyzed using 4 PVD-cyclicity categories that considered the following combinations: NPVD-CYC = absence of PVD and cycling; PVD-CYC = presence of PVD and cycling; NPVD-ANOV = absence of PVD and anovular; and PVD-ANOV = presence of PVD and anovular. Multiple logistic regression and Cox proportional regression were used for the analysis of potential associations between PVD and cyclicity categories and pregnancy at first AI (PAI1), days from calving to pregnancy, and PL at first AI. The odds (95% confidence intervals) of pregnancy increased from cows in the PVD-ANOV category (reference category) to cows in NPVD-ANOV [2.09 (1.62–2.50)], PVD-CYC [2.52 (2.02–3.14)], and NPVD-CYC [3.46 (2.84–4.23)]. Similarly, days from calving to pregnancy were less for NPVD-CYC, followed by PVD-CYC, NPVD-ANOV, and PVD-ANOV (121.4, 137.2, 137.3, and 157.4 d, respectively). On the contrary, no clear association was identified between groups and PL. The results indicate that both PVD and ANOV had a negative impact on PAI1 and days from calving to pregnancy. The results indicated a variable magnitude in the negative impact on the reproductive traits analyzed when both conditions were combined.

## Highlights

• Almost half of the study population developed PVD or was anovular at 54 days in milk.
• Occurrence of both PVD and ANOV had a significant negative impact on pregnancy at first AI and extended the time to pregnancy.
• The observed combined effects of these conditions were greater than the independent effects of PVD and ANOV alone.

## Graphical Abstract

**Figure:**
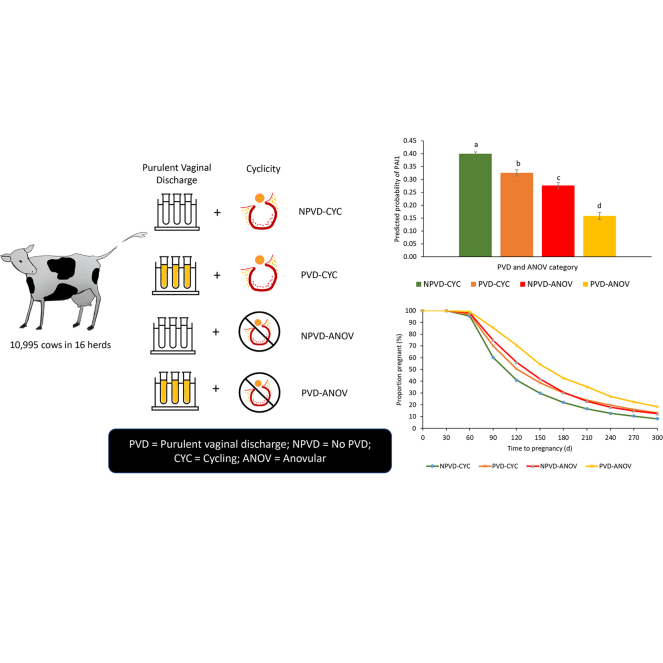
**Summary:** The objective of this study was to analyze the combined effect of purulent vaginal discharge (PVD) and anovulation (ANOV) on reproductive performance of Holstein cows. Cows were enrolled at parturition and monitored weekly for disease occurrence, reproductive events, and survival. Occurrence of PVD was assessed at 28 ± 3 days in milk and defined by the presence of mucopurulent to fetid vaginal discharge. Resumption of ovarian cyclicity was determined via transrectal ultrasonography at 40 ± 3 and 54 ± 3 d postpartum to identify anovular cows. Pregnancy diagnosis was performed by ultrasonography on d 32 ± 3 d after artificial insemination (AI) and reconfirmed at d 60 ± 3 of gestation. Pregnancy loss was defined as a cows diagnosed pregnant at 32 ± 3 but nonpregnant at 60 ± 3 d after AI. The combined effects of PVD and ANOV on pregnancy at first AI and days from calving to pregnancy were greater than the independent effects of PVD and ANOV alone.

As dairy cows transition into the lactating state, the risk for metabolic and infectious diseases increases (Drackley, 1999). Calving-associated stress, nutritional imbalances, and suppressed immunity are important factors contributing to worsened health and result in reduced milk yield, loss of body condition, and impaired fertility (Nordlund and Cook, 2004; Hammon et al., 2006).

Health conditions affecting the reproductive tract during early lactation have notorious repercussions on cow reproductive performance. Endometritis, defined as inflammation of the endometrium that occurs after the first 3 wk postpartum (Sheldon et al., 2006; Sheldon, 2020), is responsible for reducing both the risk for submission to AI and the probability of pregnancy (Runciman et al., 2008; Dubuc et al., 2011; Maquivar et al., 2015). Anovulation (**ANOV**) has also been identified as a risk factor for reduced fertility, impairing pregnancy per AI and increasing pregnancy loss (**PL**; Santos et al., 2009; Galvão et al., 2010b). The potential for a combined effect of both conditions has also been investigated. Vieira-Neto et al. (2014) evaluated the individual and combined impacts of cytological endometritis and anovulation on cow reproductive performance and determined an additive negative impact when both conditions were present. Nonetheless, to the authors' knowledge, prospective studies testing the combined effect of purulent vaginal discharge (**PVD**) and ANOV on reproductive performance are scarce. Moreover, research considering large multi-state populations and accounting for the effect of season would provide valuable information for the dairy community.

We hypothesized an additive and potentially interactive negative impact on reproductive performance when both PVD and ANOV occurred. In consequence, the objective of this study was to assess the combined effect of PVD and ANOV on the reproductive performance of a large multi-state population of Holstein cows.

The procedures performed in this research were reviewed and approved by the West Texas A&M University/Cooperative Research, Educational and Extension Team Institutional Animal Care and Use Committee (IACUC; protocol ID: 02-08-12). Farm managers also approved all of the procedures.

This study is part of a research effort investigating potential associations between genomic variation and fertility of Holstein cows (Lopes et al., 2020; Pinedo et al., 2020a,b). A convenience sample of 16 herds was enrolled based on existing contacts provided by the researchers, availability of good-quality records, daily recording of milk yield or monthly milk recording service provided by DHI, and geographic distribution across the United States. This observational prospective cohort study included 11,729 Holstein cows calving from November 2012 to October 2014 in 16 herds located in 4 regions [Northeast (4 herds), Midwest (6), Southeast (1), and the Southwest (5)]. In each herd, cows were enrolled at parturition, with half of the calvings occurring during a warm season (May to August) or a cool season (October to January) and a targeted inclusion of 33% primiparous cows. The study cows were monitored weekly for multiple reproductive events, disease occurrence, and survival. General and production cow data including parity number and monthly milk yield (DHI test day milk yield) were also collected (Pinedo et al., 2020a,b). Monitoring was performed during weekly visits to the study farms by the authors (i.e., investigator veterinarians and graduate students). Cows were inspected using headlocks or sorting gates after cows returned from the milking parlor. Prevalence of PVD was evaluated at 28 ± 3 DIM and defined by presence of mucopurulent to fetid vaginal discharge (mucus score 3 to 4; McDougall et al., 2007). Resumption of ovarian cyclicity was evaluated via transrectal ultrasonography at 40 ± 3 and 54 ± 3 DIM. Anovulation was defined as lack of visible corpus luteum on both consecutive ultrasound scans (Bicalho et al., 2008). A cyclic cow was defined as having a visible corpus luteum in at least one of the ultrasound scans. Pregnancy diagnosis was performed by ultrasonography on d 32 ± 3 d after AI and reconfirmed at d 60 ± 3 of gestation. No treatments were administered to the cows with PVD or anovular and no information regarding the occurrence of these conditions was available at the time of PVD and anovulation diagnosis. Method of AI included estrus detection (one dairy) and use of estrus synchronization protocols combined with variable levels of estrus detection (15 dairies). Pregnancy loss was defined as a cows diagnosed pregnant at 32 ± 3 but nonpregnant at 60 ± 3 d after AI. Data from the participant herds were combined in an Excel spreadsheet (Microsoft Corp.) and checked for consistency by 2 researchers. A single overall sample size determination was not performed. The multistate approach was to enroll the largest number of cows that was feasible for weekly monitoring to obtain a significant level of variation in multiple fertility variables. The post-hoc power calculation indicated power >90% when considering confidence = 95%, the current number of observations by categories, and the resulting group differences.

The association of PVD and ANOV with pregnancy traits were analyzed creating a dummy variable that considered 4 PVD-cyclicity categories that considered the following combinations: **NPVD-CYC** = absence of PVD and cycling; **PVD-CYC** = presence of PVD and cycling; **NPVD-ANOV** = absence of PVD and anovular; and **PVD-ANOV** = presence of PVD and anovular.

Only the reproductive events associated with the first AI postcalving were considered. All the analyses were completed at the cow level. Multiple logistic regression (PROC GLIMMIX; SAS release 9.4; SAS Institute Inc.) and Cox proportional regression (PROC PHREG) were used for testing potential associations between PVD-cyclicity categories and pregnancy at first insemination (**PAI1**), PL, and days to pregnancy. Variable selection was completed by backward stepwise selection. Covariables tested in the models included parity (primiparous, multiparous), season of calving (warm season = May to August; cool season = October to January), the first 3 DHI test day milk yields, and their interactions as fixed effects. Region and farm were considered as random effects in all the models. Potential interactions between PVD and cyclicity categories were subsequently tested for each of the reproductive outcomes of interest. Least squares means for days from calving to pregnancy by PVD-cyclicity categories were also calculated using ANOVA (PROC GLM). Descriptive time-to-event analysis for time to pregnancy was performed using PROC LIFETEST in SAS. From the final logistic model, predicted probabilities and 95% confidence intervals for PAI1 and PL were calculated (PROC GENMOD). Effects were considered significant when *P* ≤ 0.05. Interaction terms and controlling variables stayed in the models at *P*-value ≤ 0.10.

Overall, 10,995 cows were included in the analysis. Median (quartile 1, quartile 3) farm prevalence of PVD was 25.3% (20.1%; 31.3%), whereas mean (range by farm) prevalence was 25.7% (11.8–35.1). The mean prevalence of PVD was greater (*P* = 0.006) in multiparous (27.2%) compared with primiparous cows (24.8%). Prevalence varied (*P* < 0.0001) by region and was 24.0% (Northeast), 24.4% (Midwest), 32.9% (Southeast), and 26.4% (Southwest). Mean (range by farm) prevalence of ANOV was 28.5% (18.1–49.6) and was greater (*P* = 0.003) in primiparous (30.6%) compared with multiparous cows (27.4%). Prevalence varied (*P* < 0.0001) by region (Northeast = 27.9%, Midwest = 30.6%, Southeast = 23.6%, and Southwest = 27.4%). Cow frequencies for PVD-cyclicity categories were NPVD-CYC = 6,088 (55.4%), PVD-CYC = 1,784 (16.2%), NPVD-ANOV = 2,094 (19.1%), and PVD-ANOV = 1,029 (9.40%).

Clear differences in the odds of pregnancy at first AI were detected between postpartum PVD-cyclicity categories. The odds (95% CI) of pregnancy increased from cows in the PVD-ANOV category to cows in NPVD-ANOV, PVD-CYC, and NPVD-CYC (Table 1). Mean (SEM) PAI1 were 40.0 (0.7%), 32.6 (1.2%), 27.7 (1.1%), and 15.9 (1.3%) for NPVD-CYC, PVD-CYC, NPVD-ANOV, and PVD-ANOV cows, respectively.

**Table 1:** Adjusted odds ratios (OR) and 95% CI for pregnancy at first AI and pregnancy loss by cow health and reproductive status category^1^

Cow health and reproductive status category^2^		Pregnancy at AI1^3^		Pregnancy loss^4^	
Purulent vaginal discharge	Anovular	OR (95% CI)	*P*-value	OR (95% CI)	*P*-value
No	No	3.46 (2.84–4.23)	<0.0001	0.95 (0.55–01.63)	0.84
Yes	No	2.52 (2.02–3.14)	<0.0001	1.79 (1.00–3.20)	0.05
No	Yes	2.09 (1.62–2.50)	<0.0001	1.03 (0.81–1.31)	0.72
Yes	Yes	Referent	—	Referent	—

1 The statistical models included parity (primiparous, multiparous) and season of calving as fixed effects. Region and farm were included as random effects. Interactions tested were not significant and were removed from the models.

2 Purulent vaginal discharge was evaluated at 28 ± 3 DIM, and ovarian cyclicity was determined by transrectal ultrasonography at 40 ± 3 and 54 ± 3 d postpartum.

3 Cows were diagnosed pregnant via transrectal ultrasonography on 32 ± 3 d after AI and reconfirmed at d 60 ± 3 of gestation.

4 Pregnancy loss was defined as a cow diagnosed pregnant at 32 ± 3 d but nonpregnant at 60 ± 3 d after AI.

When considering the PVD-ANOV category as a reference, the adjusted hazard ratios of pregnancy were 1.86, 1.42, and 1.36 for NPVD-CYC, PVD-CYC, and NPVD-ANOV, respectively (*P* < 0.0001). Days from calving to pregnancy were less for NPVD-CYC, followed by PVD-CYC, NPVD-ANOV, and PVD-ANOV (Figure 1). Main effects of PVD and ANOV categories were detected (*P* < 0.01), as well as an interaction between PVD and ANOV category (*P* < 0.01). However, occurrence of both PVD and ANOV caused a greater interval to pregnancy. No significant effects of health and reproductive statuses were established on the odds of PL following AI1, considering the PVD-ANOV category as reference (Table 1). The predicted probabilities for PAI1 and PL by PVD-cyclicity category are presented in Figure 2, which provides a more intuitive interpretation of these associations.

**Figure 1:**
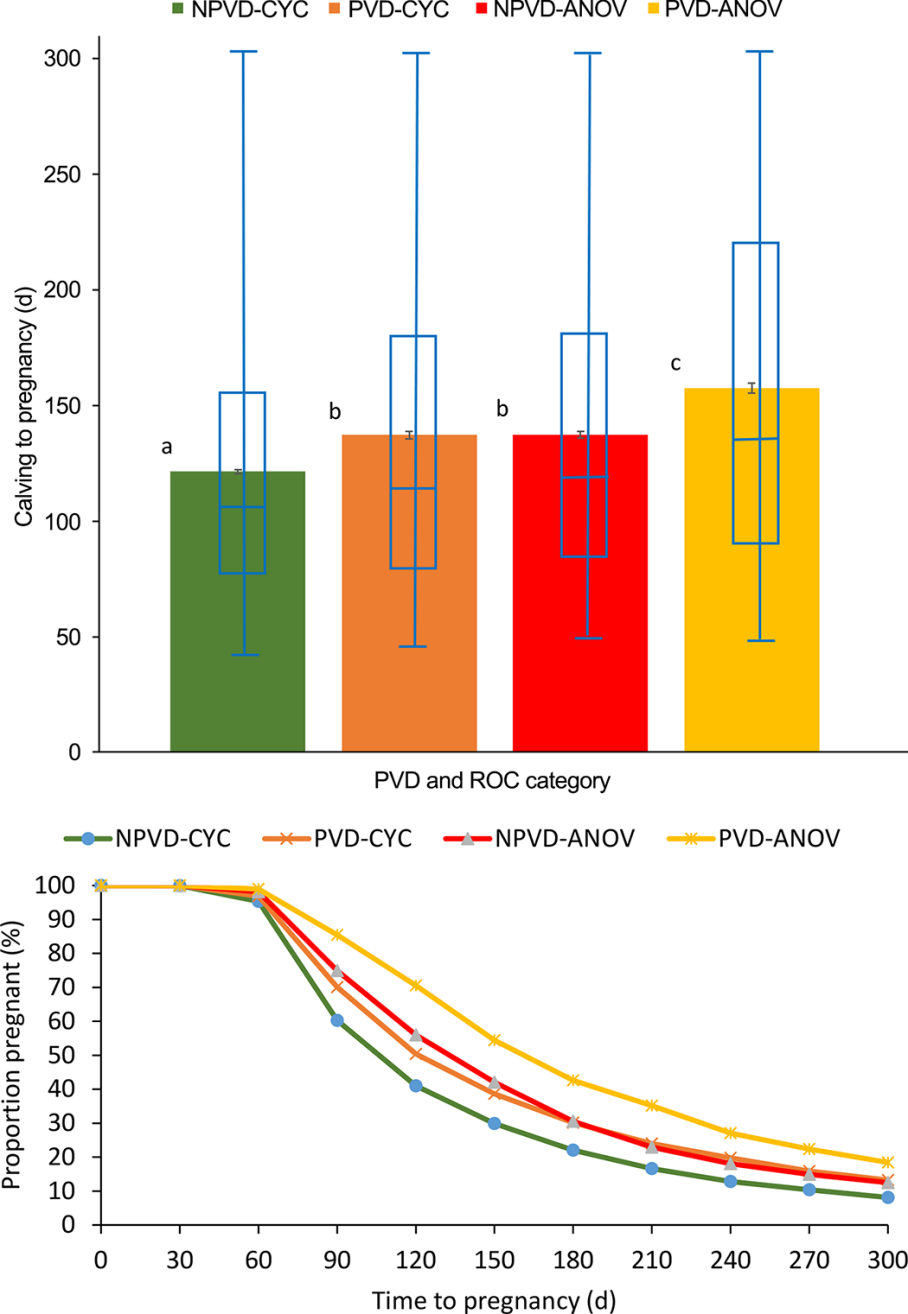
Days from calving to pregnancy [LSM (color columns) and SEM (black bars); box plot in blue; top panel] and Kaplan-Meier survival curves for the proportion of nonpregnant primiparous cows (bottom panel) by cow health and reproductive status category. Cows were diagnosed pregnant via transrectal ultrasonography on d 32 ± 3 after AI and reconfirmed at d 60 ± 3 of gestation. The box indicates quartiles 1 and 3, whereas the horizontal lines indicate minimum, median, and maximum values. Purulent vaginal discharge (PVD) was assessed at 28 ± 3 DIM, and ovarian cyclicity was determined by transrectal ultrasonography at 40 ± 3 and 54 ± 3 d postpartum. NPVD-CYC = absence of PVD (28 ± 3 DIM) and cycling (presence of a visible corpus luteum by transrectal ultrasonography at 40 ± 3 or 54 ± 3 d postpartum); PVD-CYC = presence of PVD and cycling; anovular NPVD-ANOV = absence of PVD and anovular; and PVD-ANOV = presence of PVD and anovular. ROC = resumption of ovarian cyclicity. Different letters (a–c) indicate significant differences between PVD and ROC categories (*P* < 0.05).

**Figure 2:**
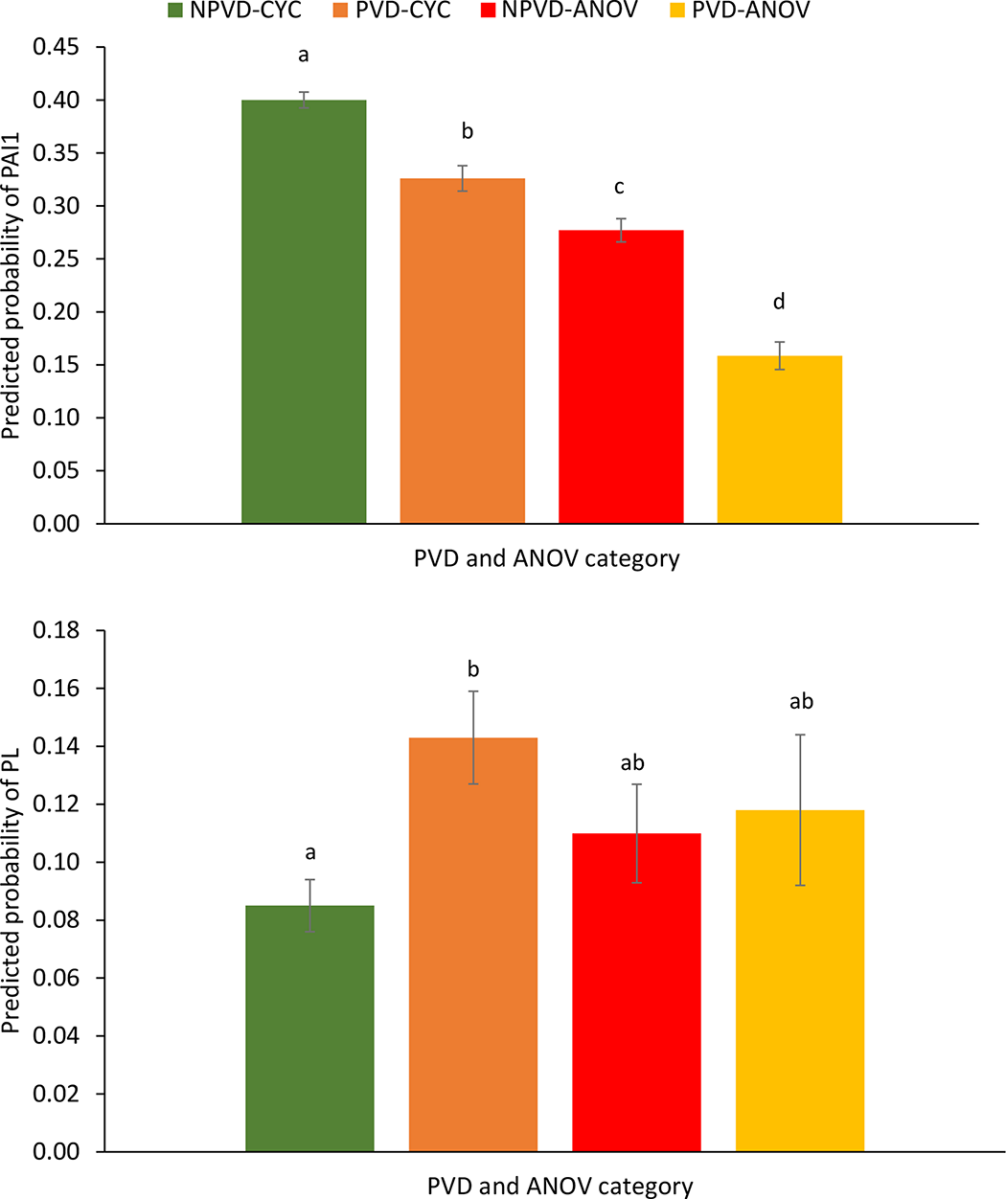
Predicted probabilities (LSM and SEM bars) for pregnancy at first AI (PAI1; top panel) and pregnancy loss (PL) at first AI (bottom panel) by cow health and reproductive status category. Cows were diagnosed pregnant via transrectal ultrasonography on d 32 ± 3 after AI and reconfirmed at d 60 ± 3 of gestation. Purulent vaginal discharge (PVD) was assessed at 28 ± 3 DIM and anestrous was determined by transrectal ultrasonography at 40 ± 3 and 54 ± 3 d postpartum. NPVD-CYC = absence of PVD (28 ± 3 DIM) and cycling (presence of a visible corpus luteum by transrectal ultrasonography at 40 ± 3 or 54 ± 3 d postpartum); PVD-CYC = presence of PVD and cycling; anovular NPVD-ANOV = absence of PVD and anovular; and PVD-ANOV = presence of PVD and anovular. Different letters (a–d) indicate significant differences between PVD and ANOV categories (*P* < 0.05).

Although the negative effects of uterine disease and delayed resumption of ovarian cyclicity on fertility are recognized, to the authors' knowledge, prospective studies testing the combined effect of purulent vaginal discharge, as a proxy for clinical endometritis, and anovulation on cow fertility are not available. Current findings represent a detailed profiling of postpartum physiological effects that affect the capacity of the cow to achieve or maintain pregnancy in large herds across 4 geographical areas and environments in North America.

The present results support the conclusions from previous reports identifying negative impacts of endometritis on reproduction (Gilbert et al., 2005; McDougall et al., 2007). The main impacts of clinical endometritis could include delayed return to cyclicity after parturition (Sheldon et al., 2002), ovulation of a smaller follicle that results in smaller corpora lutea with subsequent lower circulating concentrations of progesterone (Sheldon et al., 2002), disruption of the uterine environment (Sheldon et al., 2006), and impaired embryo development (Ribeiro et al., 2016).

The present analyses recognized the impact of anovulation on PAI1 and days open, which agrees with data previously reported (Galvão et al., 2010b; Dubuc et al., 2012). In this study, the observed combined effects of PVD and ANOV on PAI1 were greater than the independent (marginal) effects of PVD and ANOV alone. When analyzing the predicted probabilities for PAI1, values decreased by 7.4 and 12.3 percentage points for cows with PVD or anovulation compared with NPVD-CYC cows. This reduction was 24.1 percentage points when cows with combined PVD and ANOV were compared with NPVD-CYC cows (Figure 2). On the contrary, only an additive effect was established on calving to pregnancy when both conditions were combined, with differences of 15.8, 15.9, and 31.7 d calculated for PVD, ANOV, and PVD-ANOV cows, relative to NPVD-CYC cows.

Both clinical and subclinical endometritis have been identified as risk factors for anovulation (Galvão et al., 2010b; Dubuc et al., 2012; Maquivar et al., 2015). The association between endometritis and anovulation is, in part, explained by the fact that bacterial contamination of the uterus in early lactation and the resulting inflammatory process may lead to a decrease in secretion of LH (Battaglia et al., 1999), slower growth and reduced size of the first dominant follicle, and reduced follicular steroidogenesis (Sheldon et al., 2002). These characteristics affect ovulatory capacity and delay resumption of ovarian cyclicity (Kadivar et al., 2014). Another important factor that helps explain the association between endometritis and anovulation is that both are associated positively with the degree of negative nutrient balance early postpartum (Beam and Butler, 1997; Galvão et al., 2010a). Also, it is plausible to infer that many cows with endometritis likely had metritis and other clinical diseases in early lactation, which alters nutrient partition (Burciaga-Robles, 2002), increasing maintenance needs and affecting nutrient balance, which would likely delay ovulation. In consequence, cows with both PVD and ANOV could be those that had other associated diseases or more severe cases of PVD, thus partially explaining the additive effects of both conditions on subsequent pregnancy per AI and days open.

Both anovulation (Santos et al., 2009) and endometritis (Lima et al., 2013; Maquivar et al., 2015) negatively affect the progression of pregnancy in dairy cows. Nonetheless, the association between anovulation and PL has not been consistently documented in dairy cows (Santos et al., 2009) and only a few studies have evaluated the effect of endometritis on PL (Lima et al., 2013; Maquivar et al., 2015). Contrary to the effects detected between postpartum vaginal discharge and anovulation on PAI1 and days to pregnancy, a consistent effect of combined PVD and ANOV was not determined for PL in this study. Interestingly, although our study did not consider the cytological assessment of endometritis, a similar finding was reported by Vieira-Neto et al. (2014) who did not detect a significant individual or combined effect of anovulation or cytological endometritis on PL.

In this prospective study, cows diagnosed with both PVD and ANOV were associated with reduced PAI1 and extended days open. The results indicated a variable magnitude in the negative impact on the reproductive traits analyzed when both conditions were combined.
